# Effects of *Anacyclus pyrethrum* L on a mouse model of inflammatory bowel disease and its influence on intestinal barrier function and gut microbiota

**DOI:** 10.3389/fmicb.2026.1869733

**Published:** 2026-07-14

**Authors:** Jiaying Wu, Qi Wang, Xuwen Mao

**Affiliations:** 1 College of Pharmacy, Xinjiang Medical University, Urumqi, China; 2 Xinjiang Key Laboratory of Biopharmaceuticals and Medical Devices, Xinjiang Medical University, Urumqi, China; 3 Xinjiang Key Laboratory of Natural Medicines Active Components and Drug Release Technology, Xinjiang Medical University, Urumqi, China

**Keywords:** *Anacyclus pyrethrum* L, gut microbiota, IBD, intestinal barrier, metabolomics

## Abstract

**Background:**

This study investigated the protective effects of *Anacyclus pyrethrum* L. (AP) in a dextran sulfate sodium (DSS)-induced colitis mouse model and explored its potential associations with intestinal barrier function, inflammatory responses, gut microbiota, and fecal metabolic profiles.

**Methods:**

DSS-induced colitis mice were treated with different doses of AP aqueous extract. Disease activity, intestinal permeability, histopathological injury, inflammatory mediators, tight junction proteins, gut microbiota, and fecal metabolites were assessed.

**Results:**

Administration of AP mitigated clinical signs of IBD in murine models. AP suppressed the expression of the innate immune receptors TLR4 and TLR9, which are known to trigger inflammatory responses. In parallel, AP enhanced the expression of the tight junction proteins occludin and ZO-1 and was associated with restoration of intestinal barrier integrity. AP therapy was also associated with increased abundance of beneficial commensal bacterial taxa, including *Bacteroides* and *Clostridiales_unclassified*. Finally, non-targeted metabolomics analysis showed that AP intervention altered specific intestinal metabolites and affected related metabolic pathways, suggesting a multifaceted mechanism of action.

**Conclusion:**

These findings suggest that AP alleviates DSS-induced colitis in association with improved intestinal barrier function, reduced inflammatory responses, and modulation of gut microbiota composition and fecal metabolic profiles.

## 1 Introduction

Inflammatory bowel disease (IBD) is a worldwide health issue marked by persistent and recurring intestinal inflammation (Hracs et al., 2025). Key variables involved in the pathogenesis of IBD include disturbances of the gut microbiota, metabolic dysregulation, dysfunction of the intestinal barrier, and abnormal immune modulation (Lloyd-Price et al., 2019; Yan et al., 2024; Pereira et al., 2024).

The intestinal barrier serves as a vital protective layer, isolating pathogens and toxins (Peterson and Artis, 2014). During IBD progression, this barrier's dysfunction leads to reduced tight junction (TJ) proteins and increased intestinal permeability, facilitating the abnormal translocation of toxins and pathogens. This, in turn, activates the mucosal immune system and triggers a persistent inflammatory response (Chen et al., 2022; Huang et al., 2025). Closely related, the gut microbiota acts as an essential microbial barrier intimately associated with IBD pathogenesis (Xu et al., 2023; Shan et al., 2022; Ni et al., 2017). In IBD, gut microbiome dysbiosis—marked by diminished diversity and altered abundance—disrupts the microbial community structure (Vich Vila et al., 2018; Metwaly et al., 2022). Reduced populations of short-chain fatty acid (SCFA)-producing bacteria, such as *Faecalibacterium prausnitzii* and *Roseburia hominis*, lead to lower butyrate levels, a crucial energy source for colonic epithelial cells (Liu et al., 2021). Similarly, fewer tryptophan-metabolizing Lactobacillus species decrease levels of aryl hydrocarbon receptor (AhR) ligands, including indole-3-propionic acid (IPA). Limited ligand availability activates the AhR signaling pathway, further impairing the intestinal barrier (Zhou et al., 2024). The gut microbiota also directly produces and regulates diverse intestinal metabolites, which reciprocally influence the microbial microenvironment (Fan and Pedersen, 2021; Levy et al., 2015; Lavelle and Sokol, 2020). Thus, examining the local and systemic interactions among the intestinal barrier, gut microbiota, and host metabolism is essential to improve understanding of IBD pathogenesis and to advance therapeutic development.

This research examines *Anacyclus pyrethrum* L. (AP), a traditional herbal medicine derived from the dried root of AP. Previous studies have reported that AP exhibits multiple biological activities, including anti-inflammatory (Manouze et al., 2017; Rimbau et al., 1999), immunostimulatory (Bendjeddou et al., 2003; Sharma et al., 2010), and analgesic (Chen et al., 2025) effects immunostimulatory (Jawhari et al., 2020). Among these, alkylamide compounds such as pellitorine have been reported to possess anti-inflammatory potential, partly through modulation of pro-inflammatory signaling pathways, including NF-κB and MAPK (Zhang et al., 2025a). In addition, short-chain fatty acids such as isovaleric acid have been implicated in intestinal barrier regulation and immune homeostasis through GPCR-related mechanisms (Wang et al., 2023). We hypothesized that AP may alleviate IBD by improving intestinal barrier integrity, modulating gut microbiota, and affecting host metabolic profiles. To test this hypothesis, we evaluated the therapeutic effects of AP aqueous extract in a DSS-induced colitis mouse model and investigated its potential mechanisms using 16S rRNA gene sequencing and non-targeted metabolomics. We further analyzed the associations among gut microbiota, fecal metabolites, intestinal barrier function, and inflammatory markers.

## 2 Materials and methods

### 2.1 Experimental drugs

Preparation of AP aqueous extract: The plant material was pulverized and combined with purified water at a 1:10 (w/v) ratio. The combination was incubated at ambient temperature for 30 min and subsequently extracted three times via reflux. The filtrates were amalgamated and condensed at diminished tension to get a desiccated extract, which was calibrated to a crude drug concentration of 1 g/mL. The extract was re-dissolved in distilled water and diluted to 2 mg/mL to obtain the AP solution. According to previous phytochemical studies of AP roots, N-alkylamides—particularly pellitorine (deca-2E,4E-dienoic acid isobutylamide) and related isobutylamide derivatives such as anacycline—have been reported as major constituents, while inulin has also been described in pharmacognostic/review literature (Alliouche Kerboua et al., 2021; Veryser et al., 2014; Althaus et al., 2017).

Rabbit polyclonal antibodies against ZO-1 and occludin were purchased from Thermo Fisher Scientific. The rabbit polyclonal antibody against TLR4 was purchased from Novus Biologicals, and the rabbit polyclonal antibody against TLR9 was purchased from Bioss Antibodies. The HRP-conjugated goat anti-rabbit IgG secondary antibody was purchased from Beyotime Biotechnology. Dextran sulfate sodium (DSS) was procured from Shanghai Kelin Biochemical Technology Co., Ltd.; dexamethasone injection was sourced from Shanghai Modern Hason (Shangqiu) Pharmaceutical Co., Ltd.

### 2.2 Animals

Six-week-old male C57BL/6J mice (20–24 g, total *n* = 60) were obtained from the Experimental Animal Center of Xinjiang Medical University. After 1 week of acclimatization, the mice were randomly assigned to six groups, with 10 mice in each group. The experimental procedures were then initiated.

### 2.3 Establishment of IBD mouse model and drug intervention

A total of 60 6-week-old male C57BL/6J mice were randomly assigned to six groups: Control, DSS model, dexamethasone (DEX), and three AP treatment groups (AP-L, AP-M, and AP-H), with 10 mice in each group. To induce colitis, mice except those in the Control group received 3% DSS in drinking water for 10 days. Control and DSS groups received 0.2 mL of sterile water by oral gavage daily. AP-L, AP-M, and AP-H groups were given AP extract orally at 25, 50, and 100 mg/kg, respectively. The DEX group received daily intraperitoneal injections of dexamethasone at 0.4 mg/kg. Body weight, stool consistency, and fecal occult blood were recorded daily to calculate the disease activity index (DAI). The DAI score was calculated as the average of three subscores, including body weight loss, stool consistency, and bleeding, with each parameter scored from 0 to 4. Body weight loss was scored as follows: 0, no weight loss; 1, 1–5% weight loss; 2, 5–10% weight loss; 3, 10–20% weight loss; and 4, >20% weight loss. Stool consistency was scored as follows: 0, normal stool; 1, slightly loose stool; 2, loose stool; 3, very loose stool; and 4, diarrhea. Bleeding was assessed using fecal occult blood test strips according to the manufacturer's instructions. The bleeding score was assigned as follows: 0, negative; 1, weakly positive; 2, positive; 3, strongly positive; and 4, gross rectal bleeding. The final DAI score was calculated as: DAI = (body weight loss score + stool consistency score + bleeding score)/ 3.

The number of biological replicates used for each analysis was defined as follows. Unless otherwise stated, clinical parameters, including body weight, DAI, colon length, colon weight, intestinal permeability, and serum inflammatory markers, were analyzed using 10 mice per group. Gut microbiota sequencing was performed using fecal samples from 8 mice per group. H&E staining and immunohistochemistry were performed using colon tissues from 6 mice per group. For histological and immunohistochemical analyses, one representative colonic section was selected from each mouse, and five non-overlapping microscopic fields were analyzed. The mean value of the five fields was used as the final value for each mouse. Non-targeted metabolomics was performed using fecal samples from the Control, DSS, and AP-H groups, with 6 mice per group. All statistical analyses were performed using individual animals as biological replicates.

### 2.4 Intestinal permeability assessment

Upon completion of the experiment, mice were fasted for 4 h and then orally gavaged with FITC-dextran solution. FITC-dextran was prepared at a concentration of 80 mg/mL and administered at 0.1 mL/10 g body weight, corresponding to a dose of 800 mg/kg. Four hours after gavage, mice were euthanized, and blood was collected from the ocular venous plexus. The serum was isolated and fluorescence intensity was measured using a fluorescence microplate reader. A standard curve was generated using FITC-dextran standard solutions, and serum FITC-dextran concentrations were calculated accordingly.

### 2.5 Hematoxylin and eosin staining

Histopathological assessment and image acquisition were performed using a Nikon Eclipse Ni-U light microscope (Nikon, Tokyo, Japan). For each mouse, one representative H&E-stained colonic section was selected, and five non-overlapping microscopic fields were randomly captured at the same magnification. Histological evaluation was performed independently by two investigators blinded to the experimental groups. Inflammatory cell infiltration was scored using a semi-quantitative 0–4 scale: 0, no inflammatory infiltration; 1, mild infiltration mainly confined to the mucosa; 2, moderate infiltration involving the mucosa and submucosa; 3, severe infiltration extending into the muscular layer; and 4, extensive transmural infiltration with marked tissue destruction. The mean score of the five fields was used as the inflammatory infiltration score for each mouse.

### 2.6 ELISA analysis

Mouse serum was collected and tested for CXCL1, MPO, IL-6, and TNF-α levels according to the ELISA kit instructions (Nanjing Jiancheng Bioengineering Institute).

### 2.7 Immunohistochemistry

Paraffin-embedded colon tissue sections (approximately 5 μm thick) were dewaxed, rehydrated through a graded ethanol series, and subjected to heat-induced antigen retrieval in 10 mM citrate buffer (pH 6.0) by boiling for 15 min. Endogenous peroxidase activity was blocked with 3% H_2_O_2_, followed by blocking with 10% goat serum. Sections were incubated overnight at 4 °C with the following primary antibodies: rabbit anti-ZO-1 (2.5 μg/mL), rabbit anti-occludin (2.5 μg/mL), rabbit anti-TLR4 (2.5 μg/mL), and rabbit anti-TLR9 (1:200). After washing with PBS, sections were incubated with HRP-conjugated goat anti-rabbit IgG secondary antibody (1:500) for 1 h at room temperature. Color development was performed using 3,3′-diaminobenzidine (DAB), followed by counterstaining with hematoxylin, dehydration, clearing, and mounting. Immunohistochemically stained sections were digitally scanned using a Pannoramic SCAN/SCAN II whole-slide scanner. For each mouse, one representative colonic section was analyzed. Five non-overlapping fields were randomly selected from the mucosal layer. Using ImageJ software, the average optical density (AOD) of DAB-positive staining was calculated as the ratio of integrated optical density (IOD) to the positive staining area, under identical threshold settings for all images. The mean AOD value of the five fields was used as the final value for each mouse. All image analysis was performed in a blinded manner.

### 2.8 Bacterial DNA extraction

Fecal microbial genomic DNA was extracted using a CTAB-based method. Briefly, fecal samples were suspended in CTAB extraction buffer preheated to 65 °C and incubated in a 65 °C water bath for 60 min, with mixing two to three times during incubation to promote sufficient lysis. After centrifugation, the supernatant was collected and extracted with chloroform-isoamyl alcohol. DNA was precipitated using isopropanol and sodium acetate, air-dried, and dissolved in sterile ddH_2_O containing RNase. The integrity and quality of the extracted DNA were assessed by agarose gel electrophoresis, and DNA concentration and purity were measured using a NanoDrop ND-1000 spectrophotometer (NanoDrop, Wilmington, DE, USA) and a Qubit fluorometer (Invitrogen, USA) with the Qubit dsDNA HS Assay Kit (Invitrogen, Life Technologies, Q32854).

The purified DNA was used as the template for PCR amplification of the bacterial 16S rRNA gene. PCR amplification was performed using Phusion Hot Start Flex 2X Master Mix (Shanghai Yitao Biological Instrument Co., Ltd., M0536L) on an A200 thermal cycler (LongGene Scientific Instruments Co., Ltd., Hangzhou, China). PCR products were verified by 2% agarose gel electrophoresis using an electrophoresis system (Tanon EPS300, China) and visualized with a Tanon-2500 gel imaging system. The PCR products were purified using AMPure XT beads (Beckman Coulter Genomics, A63880) and quantified using a Qubit fluorometer. The purified amplicon libraries were further evaluated using an Agilent 2100 Bioanalyzer (Agilent Technologies, USA). Qualified libraries were pooled in appropriate proportions, denatured with NaOH, and subjected to paired-end sequencing on an Illumina NovaSeq 6000 platform using the NovaSeq 6000 SP Reagent Kit (500 cycles; Illumina, 20028402).

### 2.9 Illumina sequencing and data processing

The bacterial 16S rRNA gene was amplified by PCR using Phusion Hot Start Flex 2X Master Mix. Each 25 μL PCR reaction contained 12.5 μL of Phusion Hot Start Flex 2X Master Mix, 2.5 μL of forward primer, 2.5 μL of reverse primer, 50 ng of template DNA, and nuclease-free water to a final volume of 25 μL. PCR amplification was performed under the following conditions: initial denaturation at 98 °C for 30 s; 32 cycles of denaturation at 98 °C for 10 s, annealing at 54 °C for 30 s, and extension at 72 °C for 45 s; followed by a final extension at 72 °C for 10 min. PCR products were verified by 2% agarose gel electrophoresis, purified using AMPure XT beads, and quantified using a Qubit fluorometer. The purified amplicons were further assessed using an Agilent 2100 Bioanalyzer and an Illumina/Kapa library quantification kit. Qualified libraries were pooled at appropriate ratios and subjected to paired-end sequencing on the Illumina NovaSeq 6000 platform using the NovaSeq 6000 SP Reagent Kit for 2 × 250 bp sequencing. Raw paired-end reads were demultiplexed according to barcode sequences, and adapters and barcode sequences were removed. Primer sequences were removed using cutadapt, paired-end reads were merged using FLASH, and low-quality sequences were filtered using fqtrim. Chimeric sequences were removed using Vsearch. DADA2 implemented in QIIME2 was used for denoising and generation of amplicon sequence variants. Taxonomic annotation was performed using the SILVA database.

### 2.10 RNA bioinformatics data analysis

To assess species diversity within habitats, α-diversity was analyzed using observed species, Shannon, Simpson, and Chao1. Based on the obtained species abundance data, differential abundance analysis was conducted between comparison groups. The choice of statistical tests was determined by sample characteristics: Fisher's exact test was utilized for comparisons involving samples lacking biological replicates; the Mann-Whitney U test was employed for comparisons between two groups with replicates; and the Kruskal-Wallis test was applied for comparisons among multiple groups with replicates. A significance level of *P* < 0.05 was utilized.

### 2.11 Non-targeted metabolomics analysis based on UPLC-ESI-MS/MS

Approximately 100 mg of fecal sample was weighed and transferred to a centrifuge tube containing grinding beads. Subsequently, 1,000 μL of pre-chilled methanol/water (4:1, v/v) solution was added. After low-temperature ultrasonic extraction, the supernatant was collected for subsequent UPLC-MS/MS analysis. Chromatographic separation was carried out on an Acquity UPLC^®^ HSS T3 column (2.1 × 100 mm, 1.8 μm; Waters, Milford, MA, USA) with an injection volume of 1 μL. Mass spectrometric detection was performed in both positive and negative ionization modes.

### 2.12 Statistical analysis

Statistical analysis was conducted with GraphPad Prism 9.0 software. Continuous data are shown as the mean ± standard error of the mean (SEM). Categorical data are represented as percentages. Comparisons between the two groups were conducted utilizing the unpaired Student's *t-*test. One-way analysis of variance (ANOVA) was used to compare several groups. Two-way ANOVA was employed when two independent variables were present. The Kruskal–Wallis test was utilized for data that failed to satisfy normality assumptions. A *P*-value of less than 0.05 was deemed statistically significant.

## 3 Results

### 3.1 AP ameliorates disease severity in IBD mice

**Figure 1:**
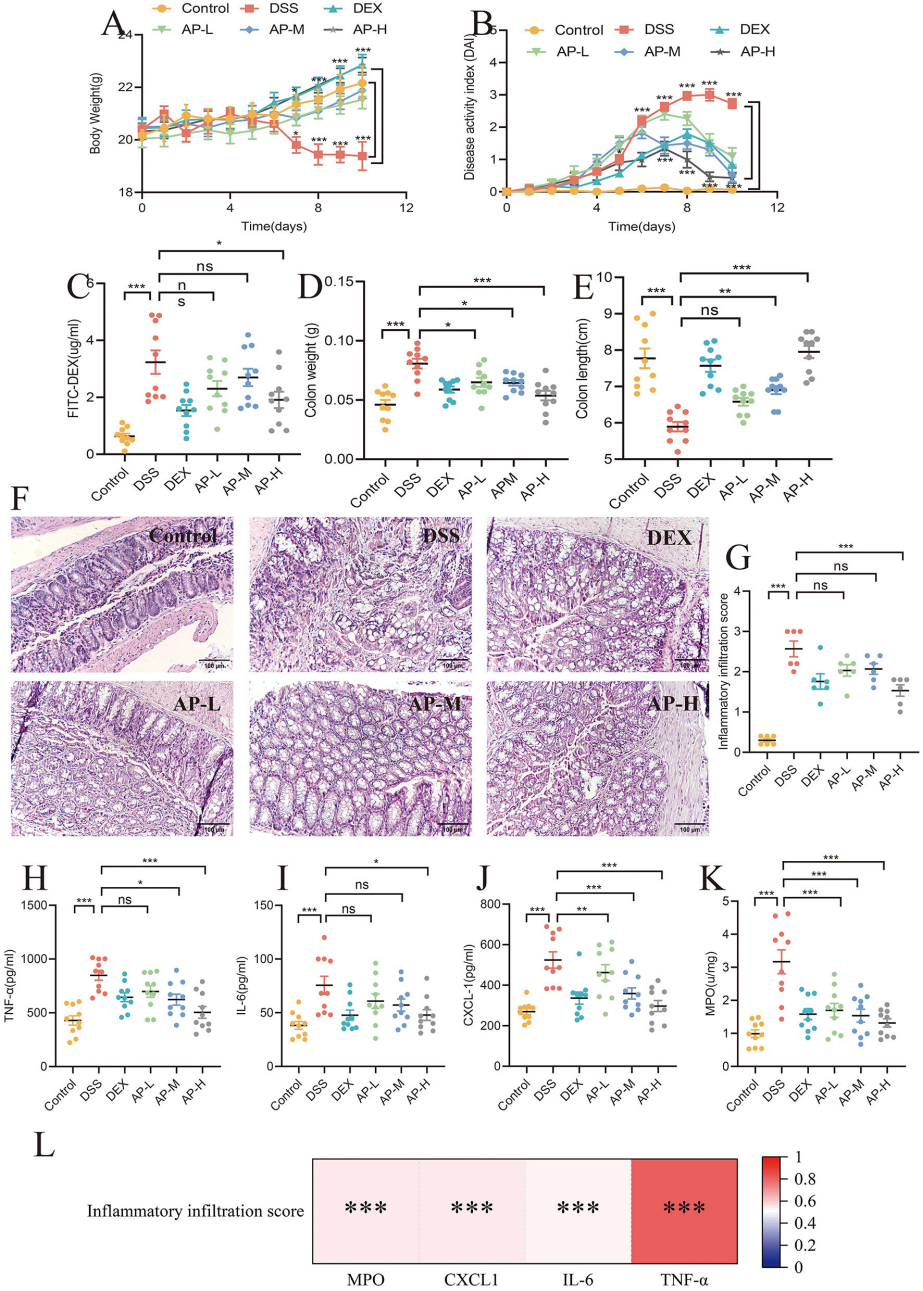
AP mitigates the inflammatory symptoms in IBD mice. **(A)** Changes in body weight over time. **(B)** Disease activity index (DAI) scores over time. **(C)** Intestinal permeability assessed by FITC-dextran. **(D)** Colon weight. **(E)** Colon length. **(F)** Representative H&E-stained colon sections from the Control, DSS, DEX, AP-L, AP-M, and AP-H groups (scale bar = 100 μm). **(G)** Semi-quantitative scores of inflammatory cell infiltration in H&E-stained colonic sections. **(H–K)** Serum levels of TNF-α, IL-6, MPO, and CXCL1 measured by ELISA. **(L)** Spearman correlation analysis between inflammatory infiltration scores and serum levels of TNF-α, IL-6, MPO, and CXCL1 based on individual mouse-level data. Data are presented as mean ± SEM. Body weight and DAI scores were analyzed using two-way ANOVA, while other group comparisons were analyzed using one-way ANOVA followed by Tukey's multiple comparisons test. Correlation analyses were performed using Spearman's rank correlation test. *P < 0.05, **P < 0.01, ***P < 0.001.

Compared with the Control group, DSS-induced mice exhibited severe IBD symptoms, including significant reductions in body weight and colon length, as well as marked increases in the disease activity index (DAI), intestinal permeability (serum FITC-dextran levels), and colon weight (all *P* < 0.001). AP administration dose-dependently ameliorated these changes (*P* < 0.05), and the therapeutic efficacy of the AP-H group was comparable to that of the DEX group, as reflected by body weight, colon length, DAI, intestinal permeability, and colon weight (Figures 1A–E). Histopathological examination further revealed severe epithelial damage, crypt destruction, and inflammatory cell infiltration in the DSS group, whereas DEX and AP treatment markedly ameliorated these pathological changes. Representative H&E-stained colonic sections from each group are shown in Figure 1F. To further evaluate histological inflammation, inflammatory cell infiltration in H&E-stained colonic sections was semi-quantitatively scored. DSS treatment markedly increased inflammatory infiltration compared with the Control group (*P* < 0.001), indicating severe histological inflammation. In contrast, AP treatment significantly reduced inflammatory infiltration scores, particularly in the AP-H group, suggesting attenuation of colonic inflammatory injury (*P* < 0.001; Figure 1G). Consistently, serum levels of the pro-inflammatory mediators TNF-α, IL-6, MPO, and CXCL1 were significantly elevated in the DSS group (*P* < 0.001; Figures 1H–K). To further explore the relationship between histological inflammation and inflammatory responses, Spearman correlation analysis was performed using individual mouse-level data. The inflammatory infiltration score was positively correlated with TNF-α, IL-6, MPO, and CXCL1 levels (Figure 1L), indicating that histological inflammatory injury was closely associated with inflammatory cytokine and chemokine responses. Both AP and DEX treatment attenuated these alterations, as reflected by improved epithelial morphology, reduced inflammatory infiltration, and decreased levels of the above pro-inflammatory mediators (*P* < 0.05). Taken together, these results indicate that AP treatment is associated with attenuation of DSS-induced colitis, as evidenced by improvements in disease-related clinical parameters, reduced intestinal permeability, alleviated histopathological injury, and decreased inflammatory mediator levels.

### 3.2 AP modulates inflammatory protein expression and is associated with improved intestinal mucosal barrier function in IBD mice

**Figure 2:**
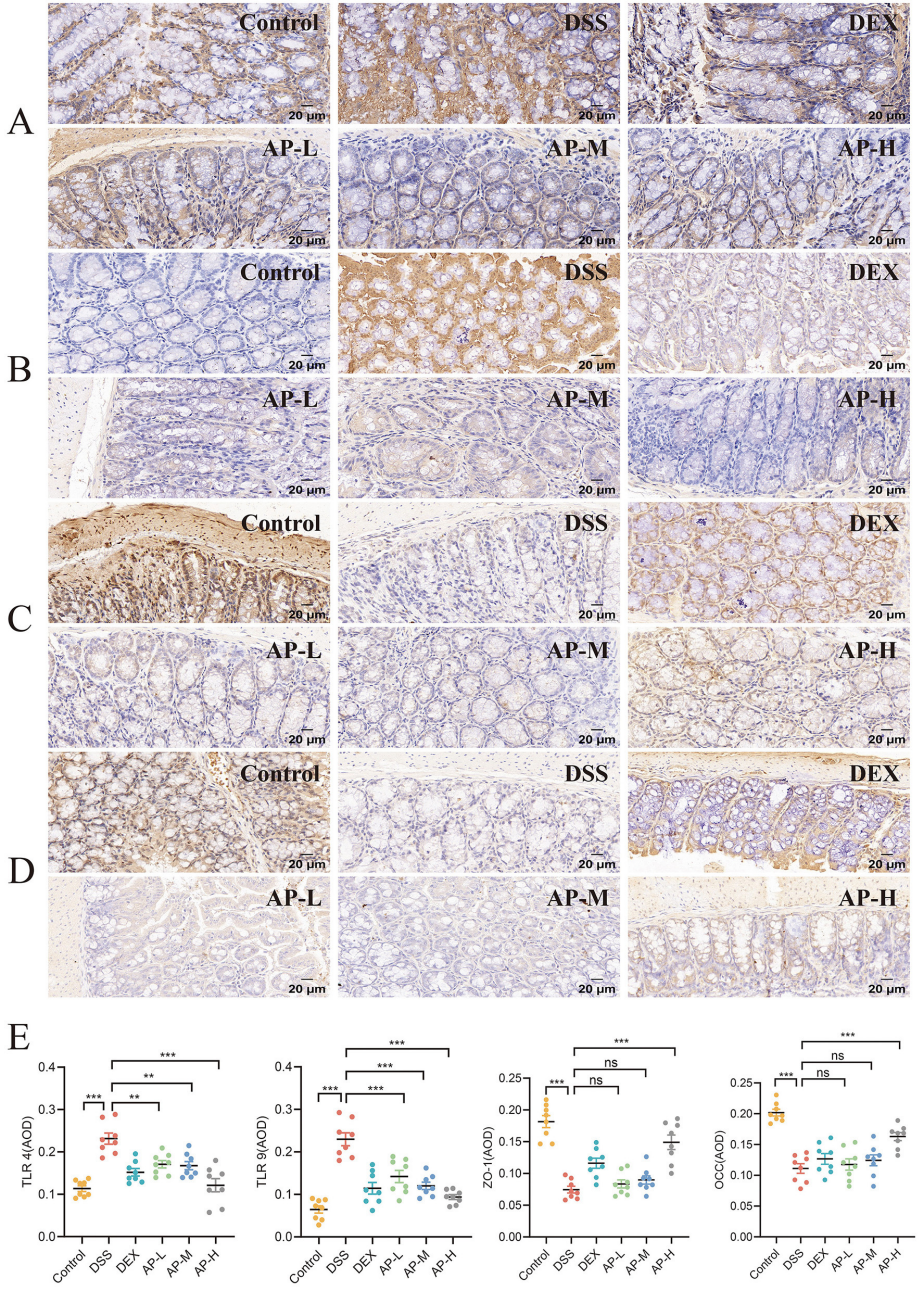
AP-modulated tight junction proteins enhance intestinal barrier function in IBD mice. **(A)** Immunohistochemical analysis of TLR4 expression in colon tissue. **(B)** Immunohistochemical analysis of TLR9 expression in colon tissue. **(C)** Immunohistochemical analysis of Occludin (OCC) expression in colon tissue. **(D)** Immunohistochemical analysis of ZO-1 expression in colon tissue. **(E)** Quantification of TLR4, TLR9, OCC, and ZO-1 expression levels. Data are presented as mean ± SEM. Statistical analysis was performed using one-way ANOVA followed by Tukey's multiple comparisons test. **P < 0.01, ***P < 0.001.

To evaluate the effects of AP on intestinal barrier-related and inflammatory proteins in colonic tissues, immunohistochemical staining and quantitative analysis were performed. Representative images of TLR4, TLR9, ZO-1, and occludin (OCC) staining are shown in Figures 2A–D, respectively. Brown-yellow staining was considered indicative of positive expression of the target proteins. The quantitative analysis indicated that TLR4 and TLR9 expression levels in the DSS group were dramatically elevated (*P* < 0.001), whereas OCC and ZO-1 expression levels were significantly decreased (*P* < 0.001). AP or DEX treatment substantially rectified the previously noted changes (*P* < 0.001, Figure 2E). These findings suggest that AP treatment was associated with decreased TLR4 and TLR9 expression and increased tight junction protein expression, which may contribute to improved intestinal barrier function.

### 3.3 AP restores gut microbial diversity and richness in IBD mice

**Figure 3:**
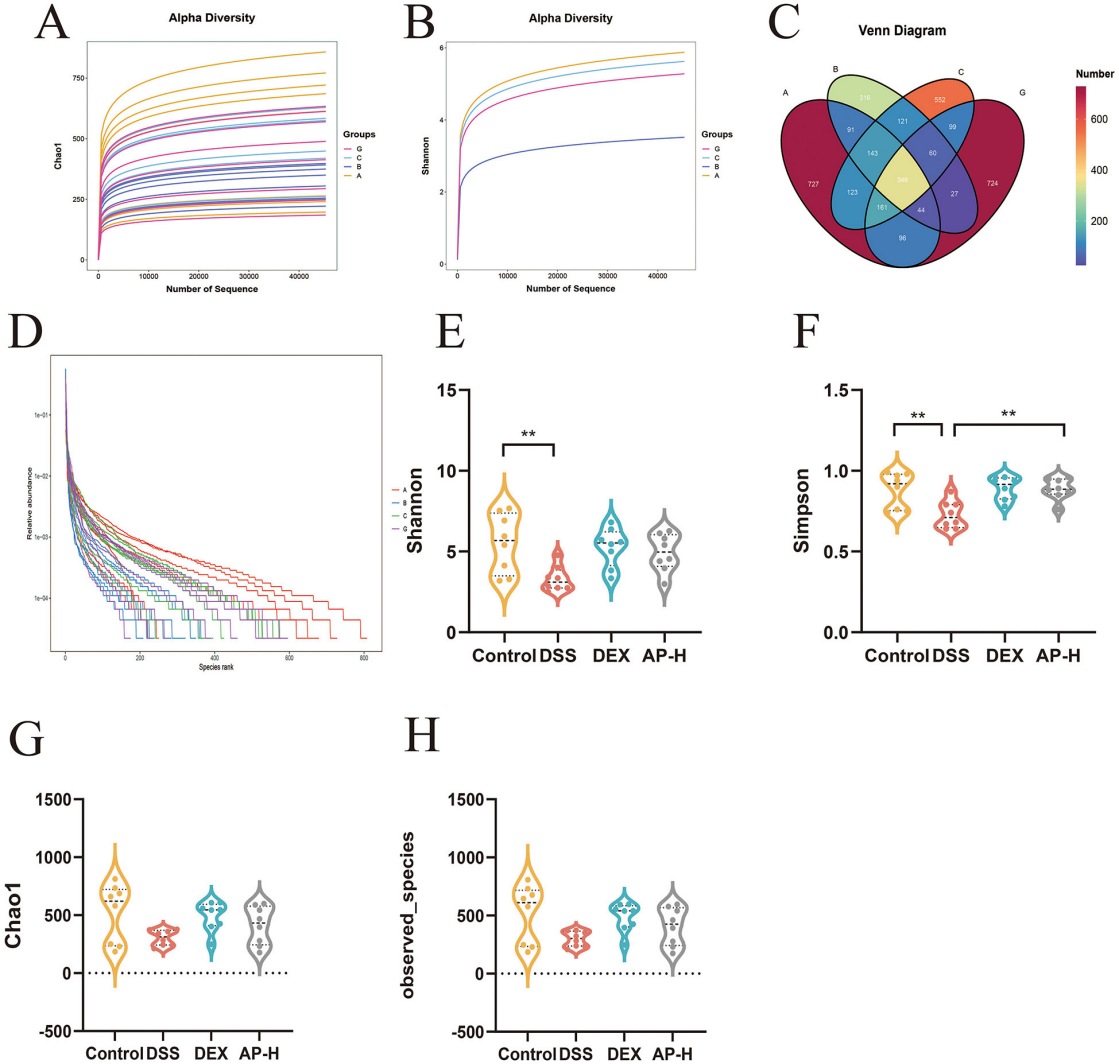
AP modulates intestinal microbiota diversity and richness in IBD mice. **(A)** Chao1 rarefaction curves. **(B)** Simpson rarefaction curves. **(C)** Venn diagrams illustrating ASV composition across groups. **(D)** Relative abundance bar plots depicting microbial community composition. **(E)** Shannon diversity index violin plots. **(F)** Simpson diversity index violin plots. **(G)** Chao1 diversity index violin plots. **(H)** Observed species richness violin plots. Data are presented as mean ± SEM. Diversity indices were analyzed using one-way ANOVA followed by Tukey's multiple comparisons test. **P < 0.01. Group labels: A, Control; B, DSS; C, DEX; G, AP-H.

16S rRNA gene sequencing was conducted to evaluate the effect of AP on the gut microbiota. The rarefaction curves demonstrated adequate sequencing depth for all samples (Figures 3A, B). Analysis of amplicon sequence variants (ASVs) revealed a significant decrease in the total ASV count in the DSS group, which was subsequently restored following treatment in both the AP-H and DEX groups (Figure 3C). Species-level relative abundance profiling indicated a rise in microbial richness in the AP-H group relative to the DSS group (Figure 3D). The assessment of α-diversity using the Shannon, Simpson, Chao1, and observed-species indices indicated a reduction in the DSS group, with partial restoration observed after the AP-H intervention (Figures 3E–H). The findings indicate that AP treatment enhanced the richness and diversity of the intestinal microbiota in IBD mice.

### 3.4 AP improves gut microbiota dysbiosis and enriches beneficial bacteria in IBD mice

**Figure 4:**
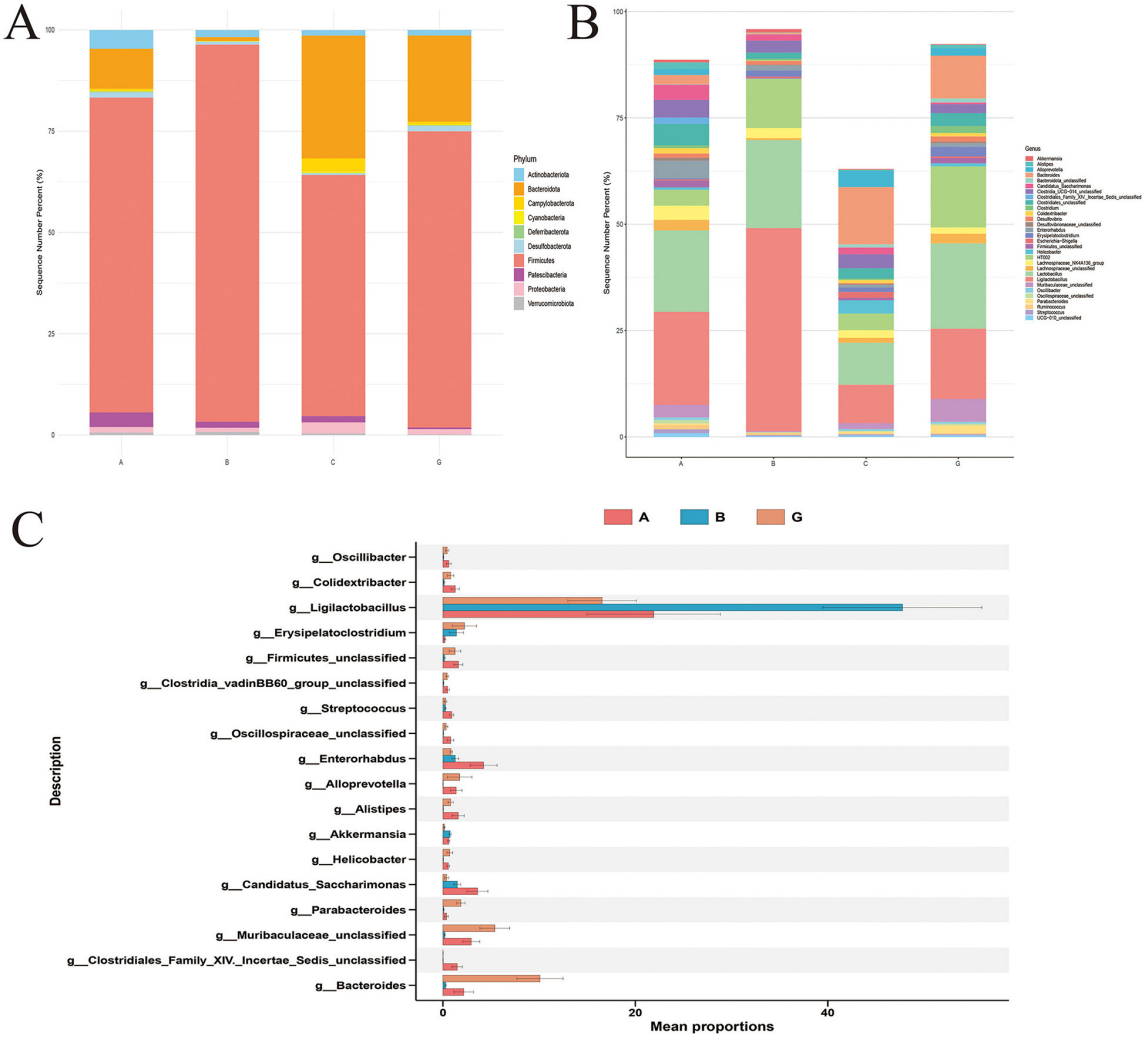
Analysis of gut microbiota composition and variations. **(A)** Bar chart showing phylum-level abundance of gut microbiota. **(B)** Bar chart illustrating genus-level abundance of gut microbiota. **(C)** Comparative analysis of differential genera among Control, DSS, and AP-H groups. Statistical analysis was performed using one-way ANOVA or Kruskal-Wallis test where appropriate. Group labels: A, Control; B, DSS; C, DEX; G, AP-H.

We investigated the microbial composition to determine how AP alters the gut microbiota in IBD mice. At the phylum level, the DSS group exhibited a notable rise in the abundance of *Firmicutes* and a reduction in *Bacteroidota* compared to the Control group. After intervention with AP-H or DEX, the elevated *Firmicutes* levels decreased, while the abundance of *Bacteroidota* returned to levels similar to those of the Control group (Figure 4A). At the genus level, the DSS group exhibited a rise in the abundance of *Ligilactobacillus*, while the abundances of *Bacteroides, Muribaculaceae_unclassified*, and *Alloprevotella* diminished. Following AP-H intervention, these changes were reversed, with the abundances of *Bacteroides* and *Muribaculaceae_unclassified* returning to levels similar to those of the Control group (Figure 4B). Genus-level differential analysis revealed that the relative abundances of many bacterial taxa, including *Bacteroides* and *unclassified Muribaculaceae*, were markedly lower in the DSS group than in the Control group (*P* < 0.05). Post-AP-H treatment, the abundances of these species markedly increased (Figure 4C; *P* < 0.05), indicating that AP increased the relative abundance of potentially beneficial bacterial taxa and was associated with improvement of microbial community structure.

**Figure 5:**
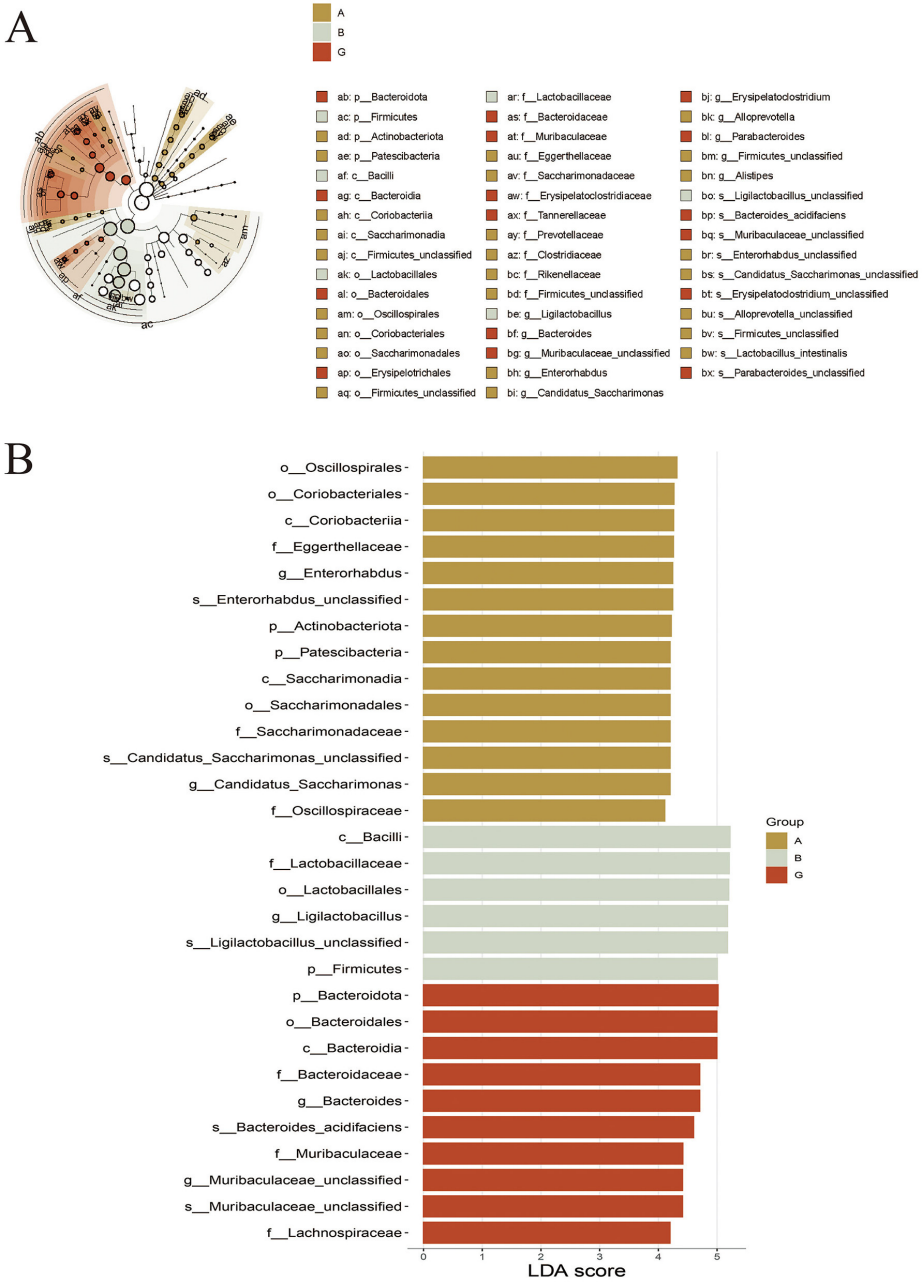
The LEfSe analysis unveiled distinct characteristics in the gut microbiota. **(A)** The evolutionary branch diagram illustrates the seven classification levels, from domain to species, using concentric circles arranged from the innermost to the outermost. Each node signifies a species at the respective taxonomic level, with node size proportional to relative abundance. Colorless nodes denote the absence of substantial differences across groups, but yellow or green nodes represent species with significant differences, with the hue denoting the group exhibiting higher relative abundance. **(B)** The LDA value distribution histogram illustrates species with statistical significance. The color corresponds to the group with higher relative abundance for the differing species, and the length of the column reflects the degree of significance in the inter-group differences. Statistical analysis was performed using the LEfSe algorithm (Kruskal-Wallis test followed by LDA). Group labels: A, Control; B, DSS; G, AP-H.

The LEfSe analysis revealed that *Bacteroides* and *Muribaculaceae_unclassified* were enriched in the AP-H group (Figures 5A, B), implying their potential role in mediating the effects of AP on IBD. These findings indicate that AP modulates the gut microbiota structure and enriches beneficial bacteria in IBD mice.

### 3.5 Associations between AP-related gut microbiota changes and pathological phenotypes in IBD mice

**Figure 6:**
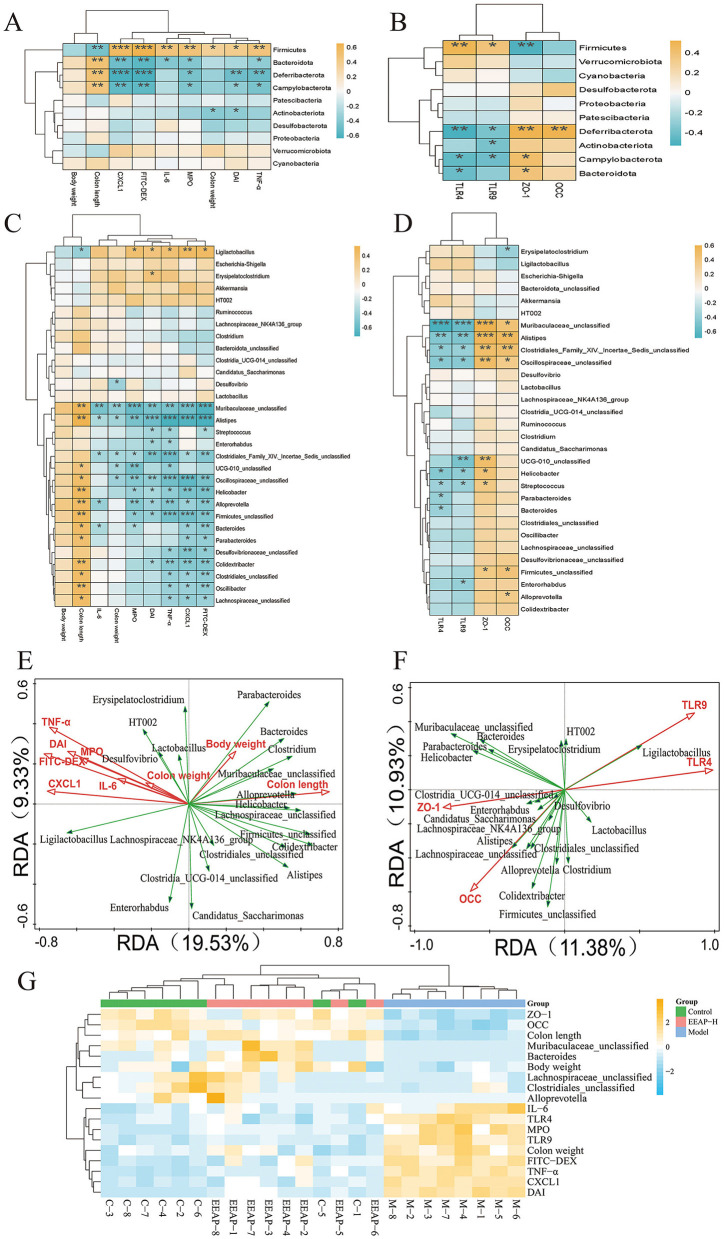
Correlation heatmap and RDA analysis. **(A)** Correlation heatmap of phylum-level gut microbiota with pro-inflammatory factors and pathological indicators. **(B)** Correlation of phylum-level gut microbiota with TLRs, OCC, and ZO-1. **(C)** Correlation heatmap of genus-level gut microbiota with pro-inflammatory factors and pathological indicators. **(D)** Correlation of genus-level gut microbiota with TLRs, OCC, and ZO-1. **(E)** RDA analysis plot illustrating the correlation between genus-level gut microbiota and pro-inflammatory factors and pathological indicators. **(F)** RDA analysis plot showing the correlation of genus-level gut microbiota with TLRs, OCC, and ZO-1. **(G)** Correlation heatmap across Control, DSS, and AP-H groups, examining associations with pro-inflammatory factors, pathological indicators, TLRs, OCC, and ZO-1. Correlation analysis was performed using Spearman correlation. *P < 0.05, **P < 0.01, ***P < 0.001.

Correlation studies were performed at the phylum and genus levels to clarify potential relationships among gut microbiota, disease phenotypes, and inflammatory markers. Distinct correlation patterns were identified at the phylum level between the two predominant phyla and several parameters in IBD mice. The relative abundance of Firmicutes was inversely associated with health indicators (body weight, colon length, TJs) and a direct relationship with disease markers (DAI, FITC-dextran, TNF-α, IL-6, CXCL1, MPO, TLR4, TLR9). In contrast, Bacteroidetes showed inverse correlations with all these variables (Figures 6A, B). At the genus level, Bacteroides, unclassified Clostridiales, unclassified Muribaculaceae, Alloprevotella, and unclassified Lachnospiraceae showed positive correlations with health indicators and negative correlations with disease indicators (Figures 6C, D). Redundancy analysis (RDA) indicates that *Bacteroides, Clostridiales_unclassified, Muribaculaceae_unclassified*, and *Alloprevotella* are positively associated with health indicators and negatively correlated with disease indicators, suggesting that these genera may serve as beneficial microbiota in mitigating IBD symptoms (Figures 6E, F). Finally, correlation analyses were conducted between each sample group and the IBD indicators. The results showed that the DSS group correlated negatively with health indicators (*Clostridiales_unclassified, Muribaculaceae_unclassified, Bacteroides, Lachnospiraceae_unclassified)*, but positively with disease indicators. Following AP-H treatment, these correlation patterns shifted toward those observed in the Control group (Figure 6G). These findings suggest that AP-H-related gut microbiota alterations were associated with increased tight junction protein expression, decreased pro-inflammatory cytokine levels, and reduced TLR expression.

### 3.6 AP treatment ameliorates fecal metabolite dysregulation in IBD mice

**Figure 7:**
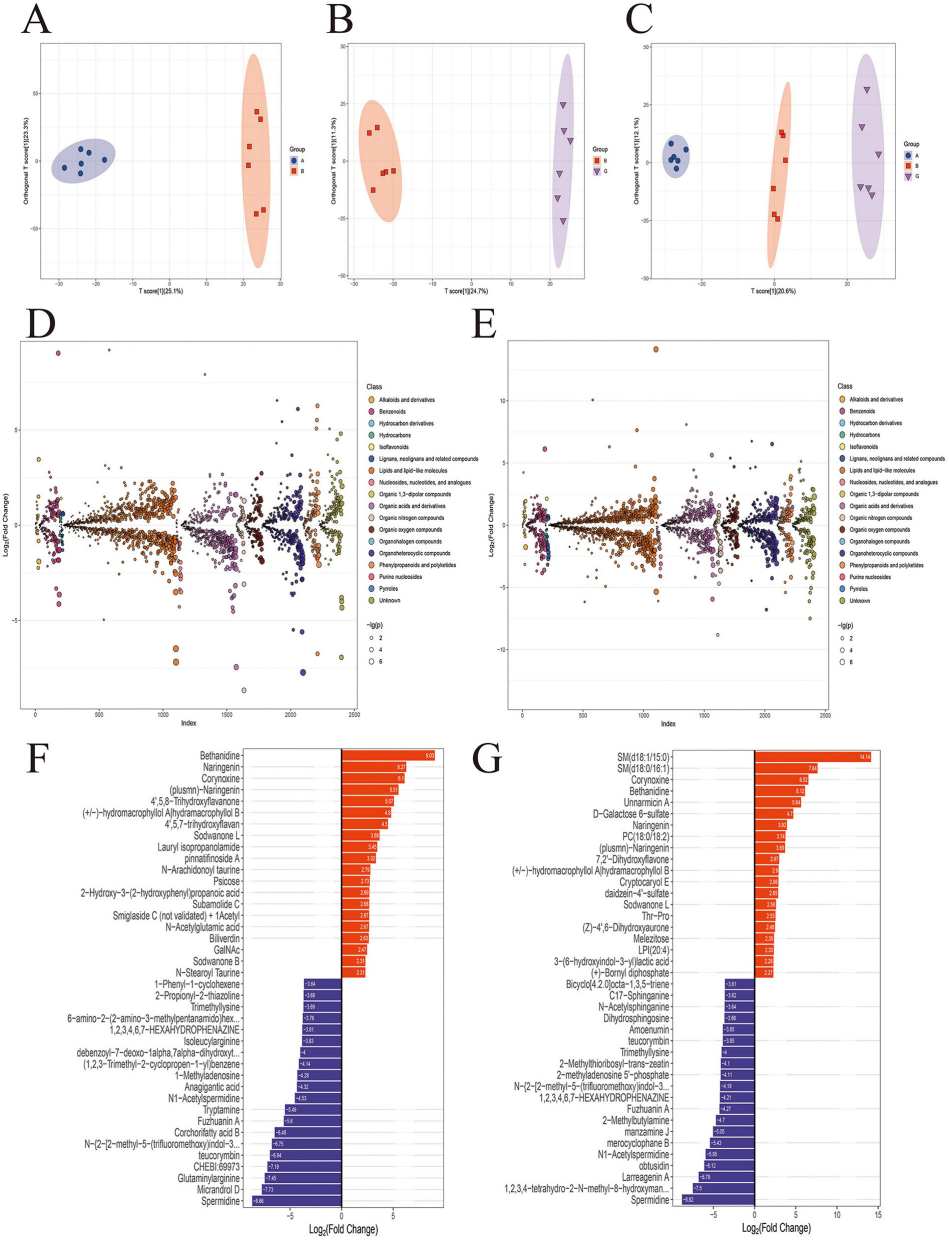
Analysis of metabolomics. **(A)** Control vs. DSS OPLS-DA analysis. **(B)** DSS vs. AP-H OPLS-DA analysis. **(C)** Control vs. DSS vs. AP-H OPLS-DA analysis. **(D)** Control vs. DSS differential metabolite volcano plot. **(E)** DSS vs. AP-H differential metabolite volcano plot. **(F)** Bar plot of top differential metabolites between Control and DSS groups based on log_2_ (Fold Change). Red indicates upregulation and blue indicates downregulation in the DSS group. **(G)** Bar plot of top differential metabolites between DSS and AP-H groups based on log_2_. Red indicates upregulation and blue indicates downregulation in the AP-H group. Multivariate statistical analysis was performed using orthogonal partial least squares-discriminant analysis (OPLS-DA). Differential metabolites were screened based on variable importance in projection (VIP > 1) combined with Student's *t-*test (*P* < 0.05). Group labels: A, Control; B, DSS; G, AP-H.

Ultimately, non-targeted metabolomic analysis was conducted to characterize fecal metabolites among experimental groups and to assess the influence of AP on the metabolic profile. OPLS-DA modeling substantially differentiated the metabolite profiles among the Control, DSS, and AP-H groups, indicating that DSS induction caused significant metabolic disruptions (*P* < 0.05), while AP-H treatment partially shifted the fecal metabolite profile toward that of the Control group (Figures 7A–C). The volcano plot analysis revealed significant differences in metabolic classes such as lipids and amino acids between the model group and the control group (*P* < 0.05), indicating that DSS treatment led to substantial changes in metabolites. Compared to the DSS group, significant changes in organic acids and nitrogen-containing compounds were observed following AP-H intervention. These results suggest that AP-H intervention may regulate specific metabolic pathways, thereby influencing the distribution and concentration of metabolites (*P* < 0.05; Figures 7D, E). Further analysis of the top 40 differential metabolites indicated that, in the Control vs. DSS comparison, 20 metabolites (including Behenidine and Naringenin) were upregulated, while 20 others (including Spermidine and Micrandrol D) were downregulated, suggesting that DSS-induced IBD substantially altered the overall fecal metabolic profile. The DSS vs. AP-H comparison showed upregulation of metabolites such as SM(d18:1/15:0) and Behenidine, and downregulation of metabolites including Spermidine and Larreagenin A, indicating widespread metabolite perturbations induced by DSS and implying a close association between metabolic state and disease pathology (Figures 7F, G). In summary, the inflammatory state induced by DSS led to extensive changes in the fecal metabolite profile, which were partially reversed by AP-H treatment, suggesting that AP may alleviate IBD by regulating specific metabolic pathways.

**Figure 8:**
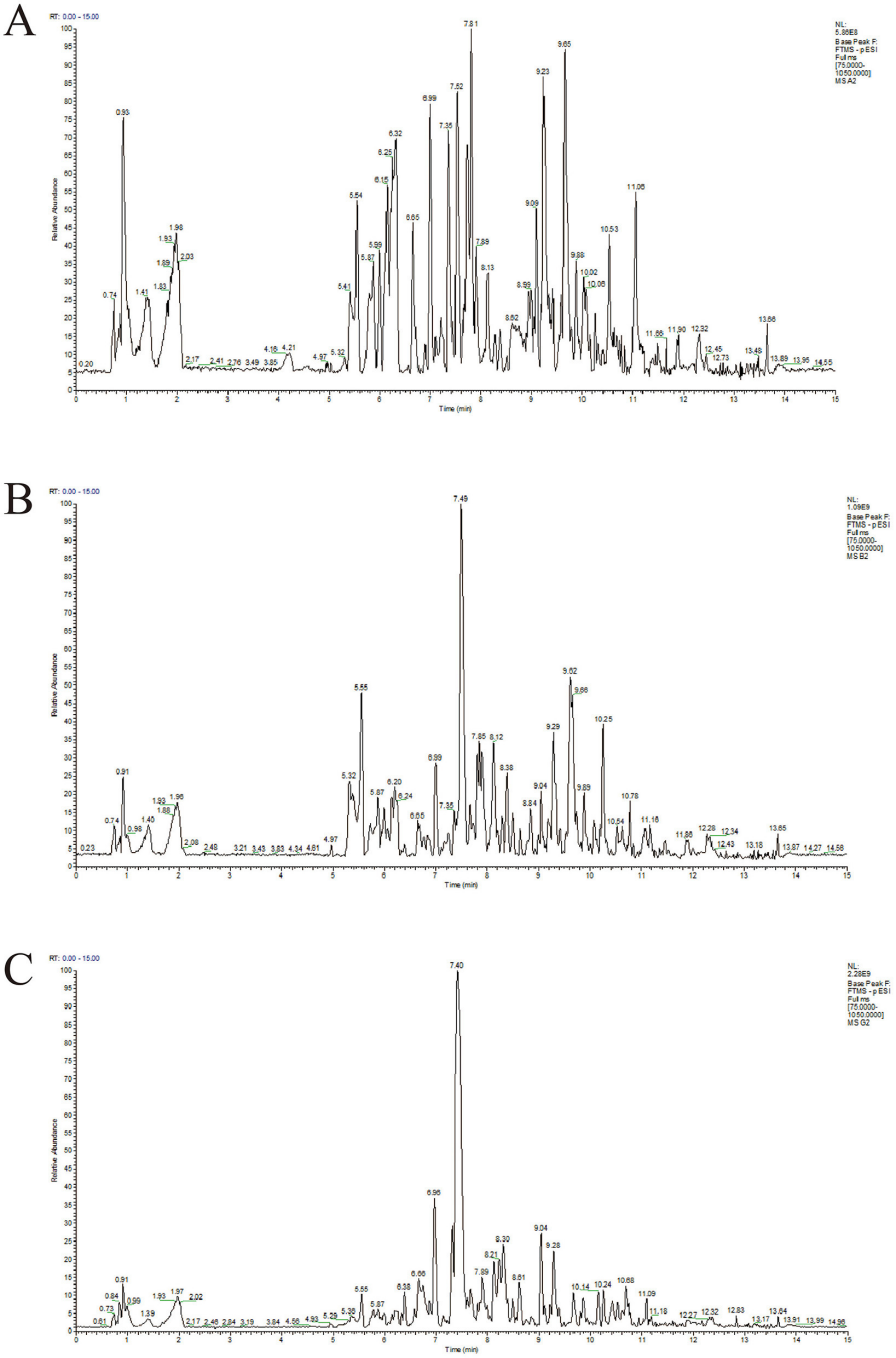
The UPLC-ESI-MS/MS figure. **(A)** Control group. **(B)** DSS group. **(C)** AP-H group.

Representative LC-MS/MS comparisons of the three sample groups are shown in Figure 8. Intersection of the differential metabolites from the two comparisons yielded six common metabolites: 3-(6-hydroxyindol-3-yl)lactic acid,2′-aminoacetophenone, dodecyl sulfate, a molecule designated as volvalerenic acid D (15-acetoxy-4-carboxy-11,11-dimethyl-bicyclogermacren-4E(5),10Z(1)-diene), 2-oxopopulifolic acid, and syringaldehyde (Table 1).

**Table 1:** Differentially expressed metabolites in the Control and DSS and AP-H group

Metabolites	m/z	Trend		VIP	*P*
		Control vs DSS	DSS vs AP-H		
3-(6-hydroxyindol-3-yl)lactic acid	220.06	UP	UP	0.55	0.000000004
2'-Aminoacetophenone	136.08	DOWN	DOWN	1.37	0.000000097
Dodecyl sulfate	265.15	UP	UP	0.88	0.000000113
15-acetoxy-4-carboxy-11,11-dimethyl-bicyclogermacren-4E(5),10Z(1)-diene|volvalerenic acid D	291.16	UP	UP	0.50	0.000000119
2-Oxopopulifolic Acid	343.22	UP	UP	0.07	0.000001614
Syringaldehyde	183.07	DOWN	DOWN	1.23	0.000001788

**Figure 9:**
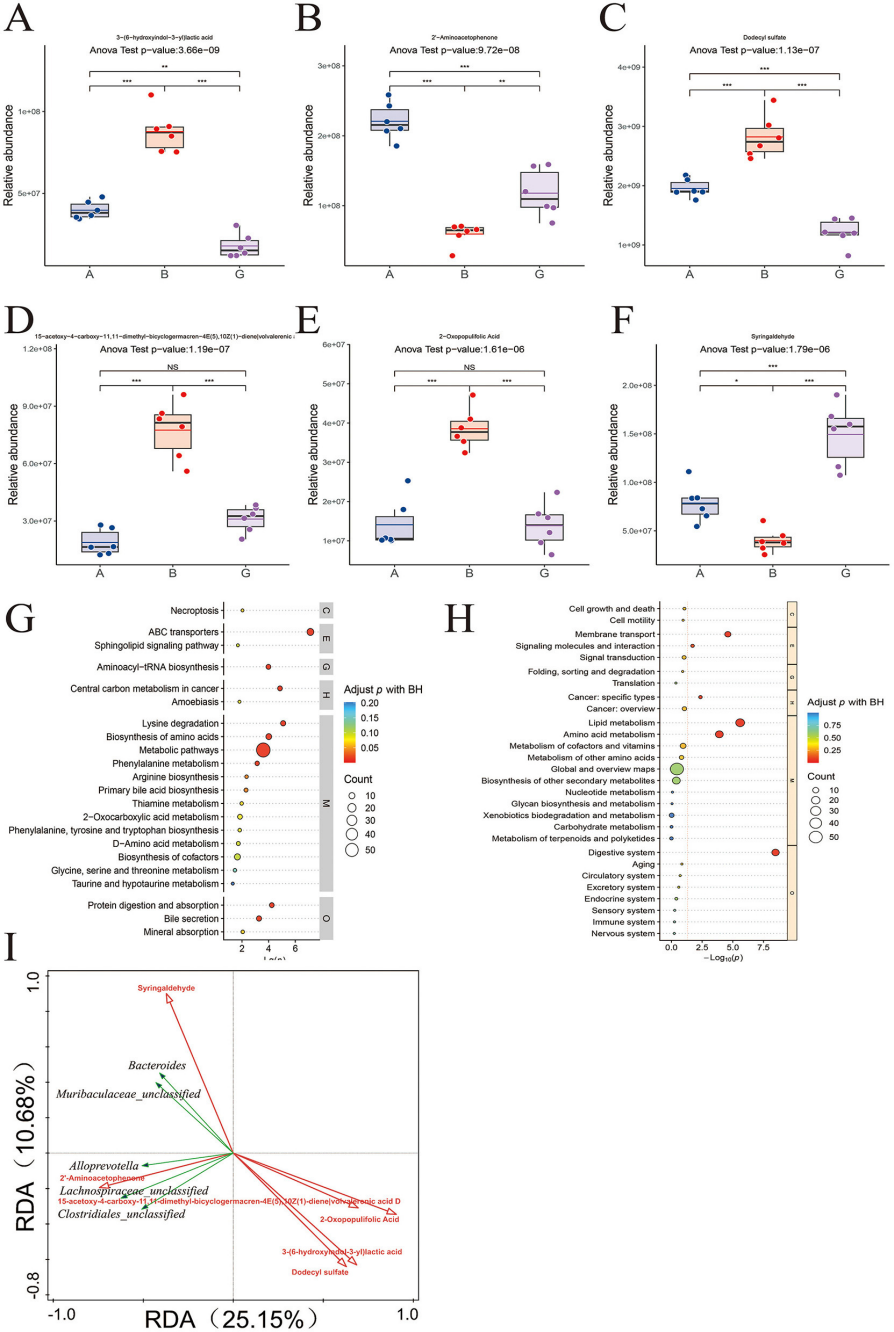
Shared differential metabolites and RDA analysis. **(A–F)** Control vs. DSS vs. AP-H comparative metabolite violin plots. **(G)** Control vs. DSS KEGG pathway analysis. **(H)** DSS vs. AP-H KEGG pathway analysis. **(I)** RDA plots of differential metabolites that are shared by the genera *Alloprevotella, Clostridiales_unclassified, Muribaculaceae_unclassified, Bacteroides*, and *Lachnospiraceae_unclassified*. Differential metabolites were identified based on variable importance in projection (VIP>1) and Student's *t-*test (*P* < 0.05). Pathway enrichment analysis was performed using KEGG database. **P < 0.01, *P < 0.05, ***P < 0.001. Group labels: A, Control; B, DSS; G, AP-H.

Analysis of differential metabolite abundance revealed that AP-H administration effectively counteracted the DSS-induced reduction in the levels of 2′-Aminoacetophenone (*P* < 0.01) and Syringaldehyde (*P* < 0.001). AP-H markedly inhibited the DSS-induced elevation of 3-(6-hydroxyindol-3-yl) lactic acid, dodecyl sulfate, volvalerenic acid D, and 2-oxopopulifolic acid (*P* < 0.001). These alterations suggest that AP-H partially reversed DSS-induced fecal metabolic disturbances (Figures 9A–F). KEGG pathway enrichment analysis revealed that the differences in metabolites between the Control and DSS groups were predominantly associated with ABC transporters and Central Carbon Metabolism. Conversely, the different metabolites between the DSS and AP-H groups were predominantly enriched in pathways associated with the digestive system and lipid metabolism (Figures 9G, H).

### 3.7 AP therapy is associated with microbiota-related changes in fecal metabolic dysregulation in IBD mice

Correlation analysis revealed associations between gut microbiota and fecal metabolites. *Bacteroides, Clostridiales_unclassified, Muribaculaceae_unclassified, Alloprevotella*, and *Lachnospiraceae_unclassified* were correlated with the fecal metabolites 3-(6-hydroxyindol-3-yl) lactic acid,2′-Aminoacetophenone, Dodecyl sulfate, volvalerenic acid D, 2-Oxopopulifolic Acid, and Syringaldehyde (Figure 9I and Table 2).

**Table 2:** Correlations between gut microbiota and serum metabolites.

Phylum	Genus	Metabolites	R	*P*
*Bacteroidota*	*Bacteroides*	3-(6-hydroxyindol-3-yl)lactic acid	−0.5060	0.0321
		2'-Aminoacetophenone	0.1739	0.4902
		Dodecyl sulfate	−0.5459	0.0191
		15-acetoxy-4-carboxy-11,11-dimethyl-bicyclogermacren-4E(5), 10Z(1)-diene|volvalerenic acid D	−0.3479	0.2302
		2-Oxopopulifolic Acid	−0.5169	0.0281
		Syringaldehyde	0.5678	0.0140
*Bacteroidota*	*Clostridiales_unclassified*	3-(6-hydroxyindol-3-yl)lactic acid	−0.1241	0.6236
		2'-Aminoacetophenone	0.4305	0.0745
		Dodecyl sulfate	−0.09000	0.7225
		15-acetoxy-4-carboxy-11,11-dimethyl-bicyclogermacren-4E(5), 10Z(1)-diene|volvalerenic acid D	−0.2435	0.3303
		2-Oxopopulifolic Acid	−0.3650	0.1364
		Syringaldehyde	−0.1072	0.6721
*Bacteroidota*	*Muribaculaceae_unclassified*	3-(6-hydroxyindol-3-yl)lactic acid	−0.6078	0.0075
		2'-Aminoacetophenone	0.2744	0.2705
		Dodecyl sulfate	−0.5231	0.0259
		15-acetoxy-4-carboxy-11,11-dimethyl-bicyclogermacren-4E(5), 10Z(1)-diene|volvalerenic acid D	−0.4923	0.0379
		2-Oxopopulifolic Acid	−0.5357	0.0220
		Syringaldehyde	0.5057	0.0323
*Bacteroidota*	*Alloprevotella*	3-(6-hydroxyindol-3-yl)lactic acid	−0.2982	0.2294
		2'-Aminoacetophenone	0.4396	0.0680
		Dodecyl sulfate	−0.2672	0.2838
		15-acetoxy-4-carboxy-11,11-dimethyl-bicyclogermacren-4E(5), 10Z(1)-diene|volvalerenic acid D	−0.3706	0.1300
		2-Oxopopulifolic Acid	−0.4561	0.0571
		Syringaldehyde	0.1285	0.6113
*Bacteroidota*	*Lachnospiraceae_unclassified*	3-(6-hydroxyindol-3-yl)lactic acid	−0.2886	0.2454
		2'-Aminoacetophenone	0.4871	0.0403
		Dodecyl sulfate	−0.2572	0.3029
		15-acetoxy-4-carboxy-11,11-dimethyl-bicyclogermacren-4E(5), 10Z(1)-diene|volvalerenic acid D	−0.3328	0.1772
		2-Oxopopulifolic Acid	−0.4498	0.0611
		Syringaldehyde	0.01045	0.9672

## 4 Discussion

In recent years, traditional Chinese medicine (TCM) has garnered significant research interest and clinical application in China, owing to its favorable safety profile and efficacy in managing IBD. In this study, we employed the TCM herb AP to treat DSS-induced colitis in mice and investigated its protective effects and potential associations with intestinal barrier integrity, gut microbiota composition, and fecal metabolic profiles.

An intact intestinal epithelial barrier constitutes the primary defense against luminal pathogens (Zhang et al., 2025b). In IBD, compromised barrier integrity facilitates the translocation of bacterial antigens, thereby activating immune responses and perpetuating inflammation (Wu et al., 2021). In accordance with this framework, our research revealed that DSS induction directly compromises the intestinal barrier, as indicated by downregulation of OCC and ZO-1, accompanied by a corresponding increase in intestinal permeability. The infiltration of gut microbiota and their metabolites into the mucosal lamina propria activates immune responses, primarily through TLR signaling pathways (Sardar et al., 2025). Excessive activation of TLR4 and TLR9 can induce the production of pro-inflammatory cytokines through downstream inflammatory signaling pathways (Zhao et al., 2025). The present study demonstrated that AP therapy markedly reduced the production of both TLRs and key pro-inflammatory mediators, such as TNF-α, IL-6, MPO, and CXCL-1, thereby mitigating DSS-induced inflammation. The gut microbiota is essential for preserving intestinal barrier homeostasis, while microbial dysbiosis significantly contributes to barrier dysfunction (Peterson and Artis, 2014; Pontarollo et al., 2023). The DSS group showed a significant reduction in the abundance of specific gut microbiota, including *Bacteroides, unclassified Clostridiales, unclassified Muribaculaceae, Alloprevotella*, and *unclassified Lachnospiraceae*. AP intervention notably restored the abundance of these microbial taxa. Among them, *Clostridiales_unclassified* is a core component of the gut microbiota; its depletion can weaken colonization resistance and predispose the gut to pathogen invasion (Sorbara and Pamer, 2022). Notably, *Bacteroides, Alloprevotella, Lachnospiraceae_unclassified*, and *Muribaculaceae_unclassified* have been reported to be associated with SCFA production in previous studies (Zaplana et al., 2024; Chen et al., 2021). SCFAs are important microbial metabolites that contribute to intestinal epithelial energy metabolism, immune homeostasis, and mucosal barrier regulation (Ambat et al., 2024; Lee et al., 2020).

The disruption of the intestinal microenvironment is closely associated with the pathological progression of IBD (Pontarollo et al., 2023; Wu et al., 2024). In the present study, DSS-induced mice exhibited a significant reduction in gut microbiota α-diversity, indicating decreased microbial richness and evenness as well as impaired ecological stability (Ni et al., 2017; Fassarella et al., 2021). In contrast, AP-H treatment partially restored intestinal microbiota diversity and increased the abundance of several potentially beneficial bacterial taxa, including *Bacteroides, Alloprevotella, Lachnospiraceae_unclassified*, and *Muribaculaceae_unclassified*. These taxa have been reported to be associated with SCFA production (Lee et al., 2020; Wu et al., 2024; Fassarella et al., 2021). SCFAs are important microbial metabolites that participate in intestinal epithelial energy metabolism, immune homeostasis, mucus production, and mucosal barrier regulation by activating G protein-coupled receptors, modulating NF-κB signaling, promoting anti-inflammatory cytokine responses, and supporting epithelial barrier integrity (Sun et al., 2018; Tran et al., 2025; Duan et al., 2023; Jiao et al., 2023). Therefore, the depletion of these potentially SCFA-associated taxa in DSS-induced mice may reflect a microbial environment unfavorable for epithelial barrier maintenance, whereas their restoration after AP-H treatment suggests a microbiota-associated shift beneficial for intestinal barrier protection. Importantly, AP-H treatment also improved barrier-related indicators, as evidenced by increased ZO-1 and OCC expression and reduced intestinal permeability. Correlation analysis further showed that these potentially beneficial bacterial taxa were positively associated with ZO-1 and OCC and negatively associated with intestinal permeability and inflammatory markers, suggesting that AP-H-induced microbiota remodeling is associated with improved intestinal barrier function. Nevertheless, acetate, propionate, and butyrate were not directly quantified in the present study, and goblet cell-derived mucins, such as MUC2, as well as mucus layer integrity, were not evaluated. Thus, the potential role of SCFA-mediated mucin production in AP-H-mediated barrier protection should be interpreted as an indirect inference based on microbiota composition and barrier-related indicators rather than direct mechanistic evidence. Future studies combining targeted SCFA quantification, mucus layer assessment, and microbiota-intervention experiments are warranted to determine whether AP-regulated microbial communities directly contribute to mucin production and intestinal barrier repair.

The gut microbiota contributes to intestinal homeostasis partly through the regulation of metabolite profiles. Non-targeted metabolomics suggested that AP-H treatment partially restored DSS-induced fecal metabolic disturbances. Among the common differential metabolites, 2′-aminoacetophenone and syringaldehyde showed relatively clearer associations with inflammatory regulation. 2′-Aminoacetophenone is a quorum-sensing-related small molecule. Previous studies have reported that it can promote host tolerance to high bacterial burden and regulate the expression and activity of HDAC1, leading to reduced H3K18 acetylation at pro-inflammatory cytokine gene loci and subsequent epigenetic reprogramming of immune responses (Bandyopadhaya et al., 2016). In addition, the interaction between NF-κB p50 and HDAC1 has also been implicated in 2′-aminoacetophenone-mediated immune tolerance (Bandyopadhaya et al., 2017). Therefore, the increased level of 2′-aminoacetophenone after AP-H treatment may indicate a shift of the intestinal metabolic environment toward immune tolerance or inflammation resolution. However, this evidence is mainly derived from studies on bacterial infection and host immune tolerance, rather than from DSS-induced colitis models. Its specific role in AP-mediated improvement of colitis therefore requires further experimental validation. Syringaldehyde is a naturally occurring phenolic aldehyde with antioxidant and anti-inflammatory activities. Previous studies have shown that syringaldehyde can ameliorate arthritis in mice by inhibiting dendritic cell maturation and pro-inflammatory cytokine secretion. It can also reduce Th1, Th17, and Th17/Th1-like immune responses while promoting Treg generation (Wen et al., 2024; Wu et al., 2022; Li et al., 2023; Mukherjee et al., 2024), suggesting that it may exert immunomodulatory effects through the dendritic cell–T cell axis. In addition, other oxidative stress- and inflammation-related models have indicated that syringaldehyde can attenuate inflammatory injury and reduce oxidative stress responses (Mortazavi Farsani and Verma, 2023; Shahzad et al., 2018, 2020). These findings are consistent with the decreased TNF-α and IL-6 levels and improved intestinal barrier function observed after AP-H treatment in the present study. Therefore, the restoration of syringaldehyde may reflect a shift of the intestinal metabolic environment toward a less inflammatory and more barrier-protective state after AP treatment. Taken together, the observed alterations in 2′-aminoacetophenone and syringaldehyde may represent potential candidate metabolic indicators associated with the alleviation of intestinal inflammation and the restoration of the microbiota–metabolite environment following AP treatment. However, their predictive value and mechanistic relevance require further validation in future studies. Meanwhile, AP-H treatment also reduced the abundance of 3-(6-hydroxyindol-3-yl) lactic acid, dodecyl sulfate, volvalerenic acid D, and 2-oxopopulifolic acid, suggesting that AP-H exerted a broad regulatory effect on DSS-induced metabolic disturbances. It should be noted that dodecyl sulfate is not a typical endogenous intestinal metabolite. In the present study, it should therefore be interpreted cautiously as an annotated differential feature detected by non-targeted metabolomics, rather than as direct evidence that AP regulates an endogenous metabolic pathway. For 3-(6-hydroxyindol-3-yl) lactic acid, volvalerenic acid D, and 2-oxopopulifolic acid, their direct biological roles in IBD remain unclear. Nevertheless, these metabolites were altered after DSS induction and partially reversed following AP-H treatment, suggesting that they may reflect inflammation status, gut microbiota dysbiosis, or AP-associated metabolic remodeling. Thus, they may serve as candidate metabolic indicators worthy of further investigation. In addition, AP-H intervention enriched several potentially beneficial bacterial taxa, including *Bacteroides, Muribaculaceae_unclassified, Alloprevotella, Clostridiales_unclassified*, and *Lachnospiraceae_unclassified*. These taxa were correlated with the above differential metabolites, indicating that AP may regulate the fecal metabolic profile partly through remodeling the gut microbiota. Previous studies have shown that microbiota-derived metabolites, particularly short-chain fatty acids, bile acids, and tryptophan-related metabolites, participate in intestinal immune homeostasis, epithelial barrier protection, and IBD progression. Therefore, AP treatment may be associated with coordinated changes in the gut microbiota–metabolite–intestinal barrier axis, which may contribute to the alleviation of DSS-induced colitis. However, because the present study was based on non-targeted metabolomics, the causal roles of individual metabolites still require further validation through targeted metabolomics, chemical characterization of AP, pharmacokinetic analysis, and microbiota-intervention experiments.

Pathway enrichment analysis indicated that, relative to the Control group, metabolites in the DSS group exhibited high enrichment in pathways such as Central Carbon Metabolism. Disruption of this system may lead to the accumulation of critical metabolites, including lactate and succinate. These metabolites are recognized for their indirect facilitation of pro-inflammatory cytokine release, such as IL-6, through the modulation of host immunological function, therefore fostering a chronic inflammatory condition (Mortazavi Farsani and Verma, 2023; Rahman et al., 2020; Gan et al., 2024). The observations suggest that the metabolic disturbance in the DSS group contributes to the dysregulation of Central Carbon Metabolism, potentially promoting the progression of intestinal inflammation to a chronic state. The comparison between the DSS and AP-H groups revealed that different metabolites were primarily enriched in pathways related to the Digestive System, particularly bile acid metabolism. Bile acids influence the integrity of the intestinal epithelial barrier and inflammatory responses by activating receptors such as FXR and TGR5 (Fiorucci et al., 2021; Pavlidis et al., 2015; Liu et al., 2022). Evidence indicates that *Bacteroides*, a genus whose prevalence increased in our study after AP therapy, produces bile salt hydrolase (BSH), an enzyme that hydrolyzes bile salts and thus alters the composition of the intestinal bile acid pool (Sun et al., 2023; Chen et al., 2024). These findings suggest that AP-related enrichment of *Bacteroides* may be associated with changes in bile acid-related metabolic pathways. However, because bile acid species and bile acid receptor signaling were not directly measured, this hypothesis requires further validation by targeted bile acid profiling and functional experiments.

Although the present study demonstrated that AP aqueous extract alleviated DSS-induced colitis and was associated with improvements in gut microbiota composition, intestinal barrier function, and fecal metabolic profiles, several limitations should be acknowledged. First, the chemical composition of the AP aqueous extract was not systematically characterized. Although the extract was prepared using a standardized procedure, UPLC-MS/MS analysis was not performed for the specific batch used in this study. Therefore, the exact constituents and their concentrations remain unclear, which may affect reproducibility and quality control. Future studies should establish a UPLC-MS/MS-based chemical fingerprint of AP aqueous extract and quantify representative candidate active constituents, such as alkylamides, amides, alkaloids, and organic acids, to clarify the chemical basis underlying its biological effects. Second, the pharmacokinetic behavior and tissue exposure of major or representative AP constituents were not evaluated. Because AP was administered orally, it remains unclear whether its constituents were absorbed into the circulation, accumulated in colonic tissue, or mainly retained within the intestinal lumen. Therefore, the present study cannot determine whether AP directly acts on intestinal epithelial or immune cells, or whether its protective effects are primarily mediated through modulation of the gut microbiota and fecal metabolites. Future studies integrating UPLC-MS/MS-based chemical profiling with pharmacokinetic and tissue distribution analyses in serum, colonic tissue, intestinal contents, and feces are needed to clarify the exposure–effect relationship of AP constituents. Third, fecal microbial DNA was extracted using a CTAB-based protocol without lysozyme or proteinase K pretreatment. This approach may have limited lysis efficiency for some Gram-positive bacteria with thick cell walls and may therefore introduce extraction-related bias in microbiota profiling. Future studies should use optimized DNA extraction protocols incorporating enzymatic lysis and/or mechanical bead-beating to improve the recovery of Gram-positive bacterial DNA.

## 5 Conclusion

This study provides evidence that AP aqueous extract alleviates DSS-induced colitis in mice and is associated with improved intestinal barrier function, reduced inflammatory responses, altered gut microbiota composition, and changes in fecal metabolic profiles. However, the chemical composition of the specific AP aqueous extract used in this study was not directly characterized, and the exact active constituents responsible for these effects remain unclear. Further mechanistic and pharmacokinetic studies are needed to validate the causal pathways underlying the protective effects of AP.

## Data Availability

The raw sequencing data are available in the NCBI SRA (https://www.ncbi.nlm.nih.gov/sra), under BioProject accession PRJNA1482255.
